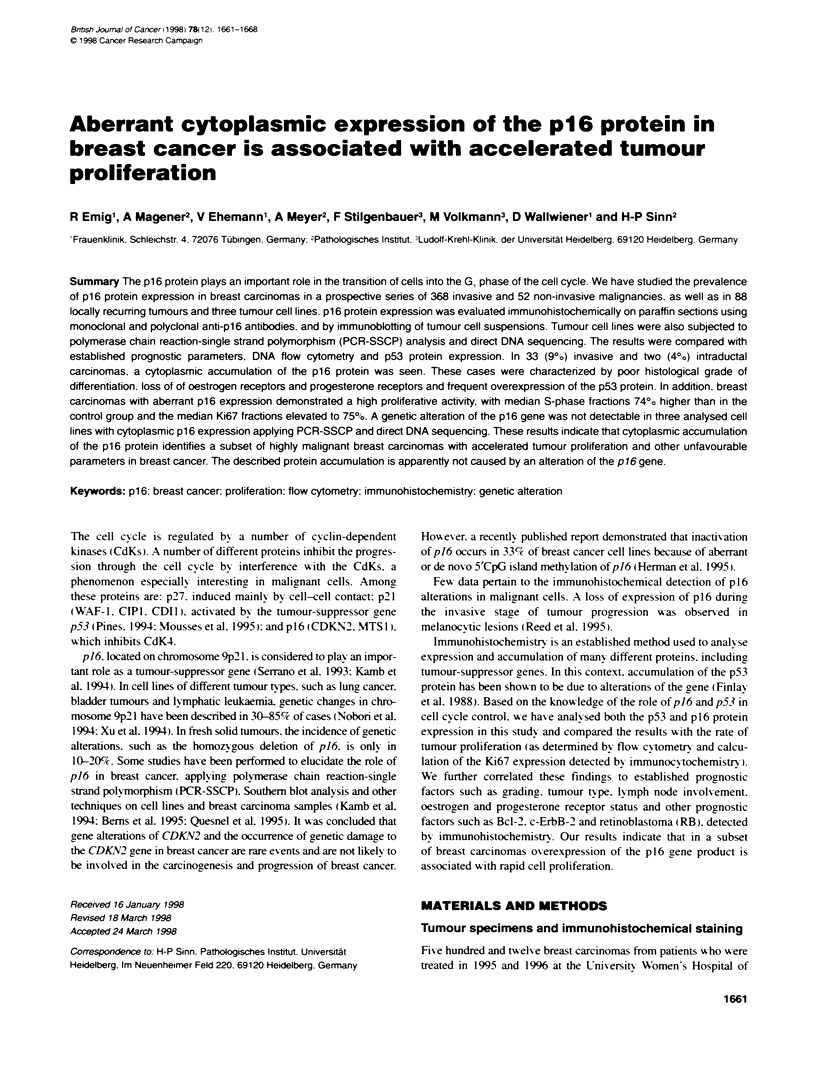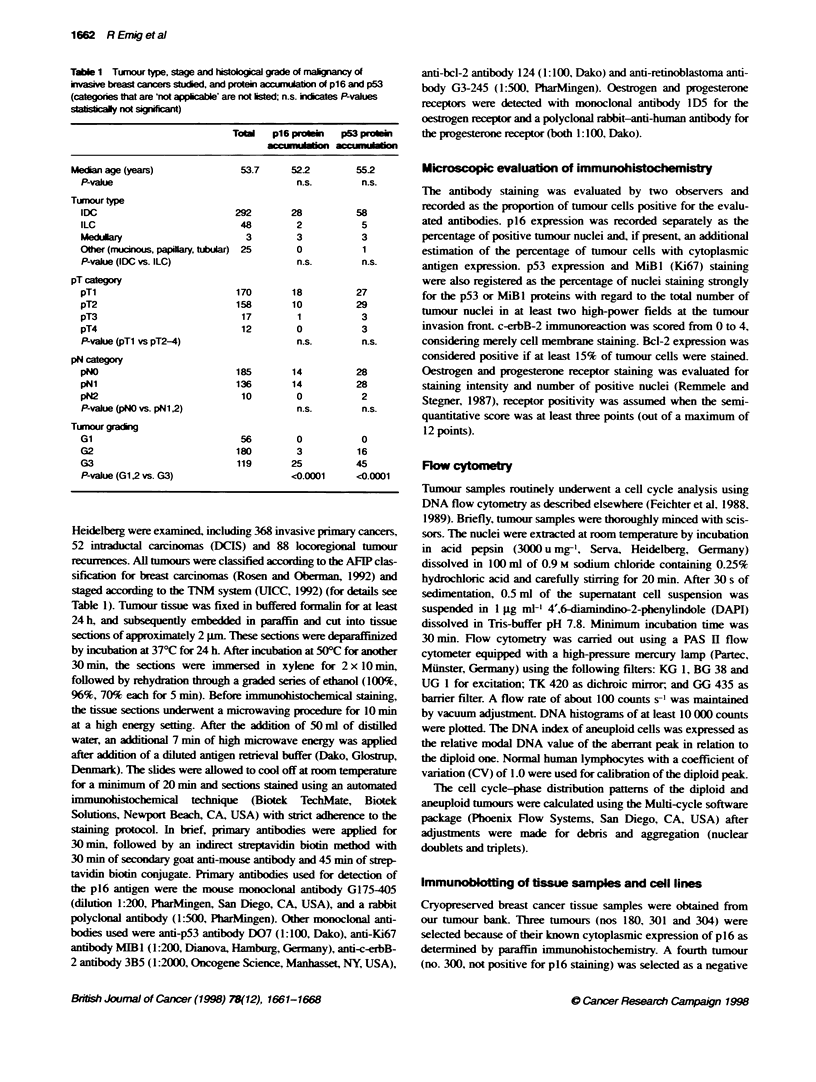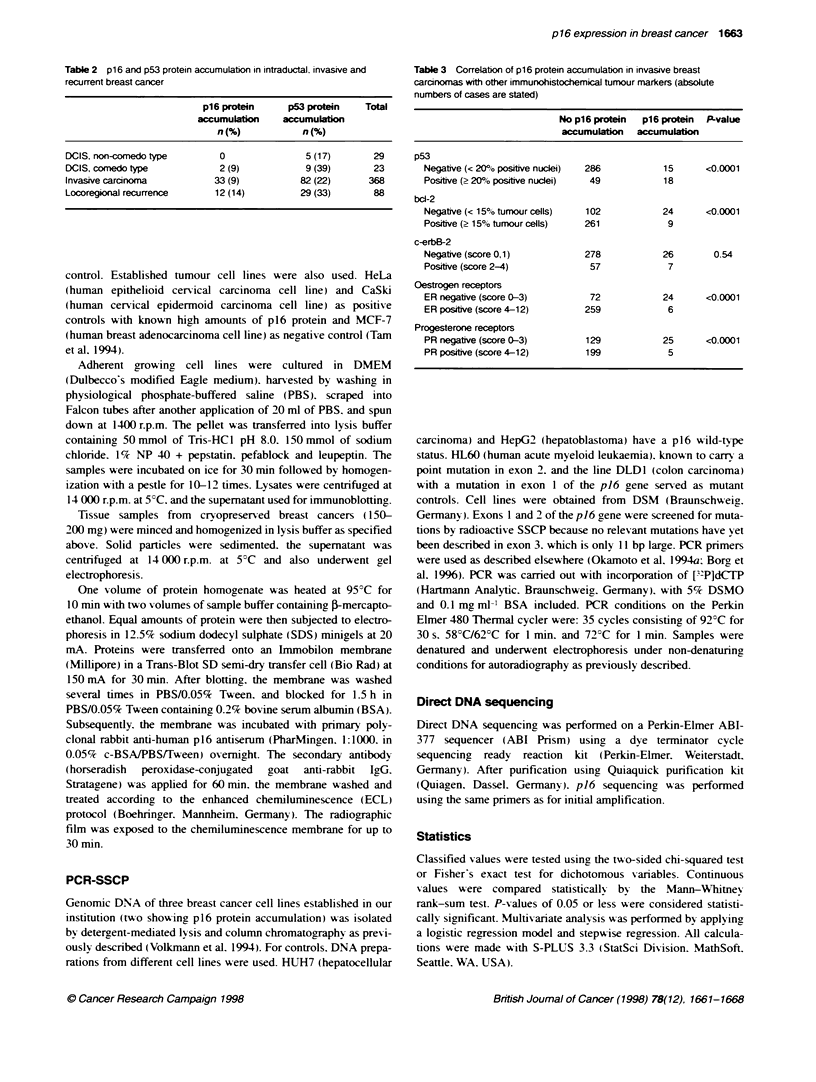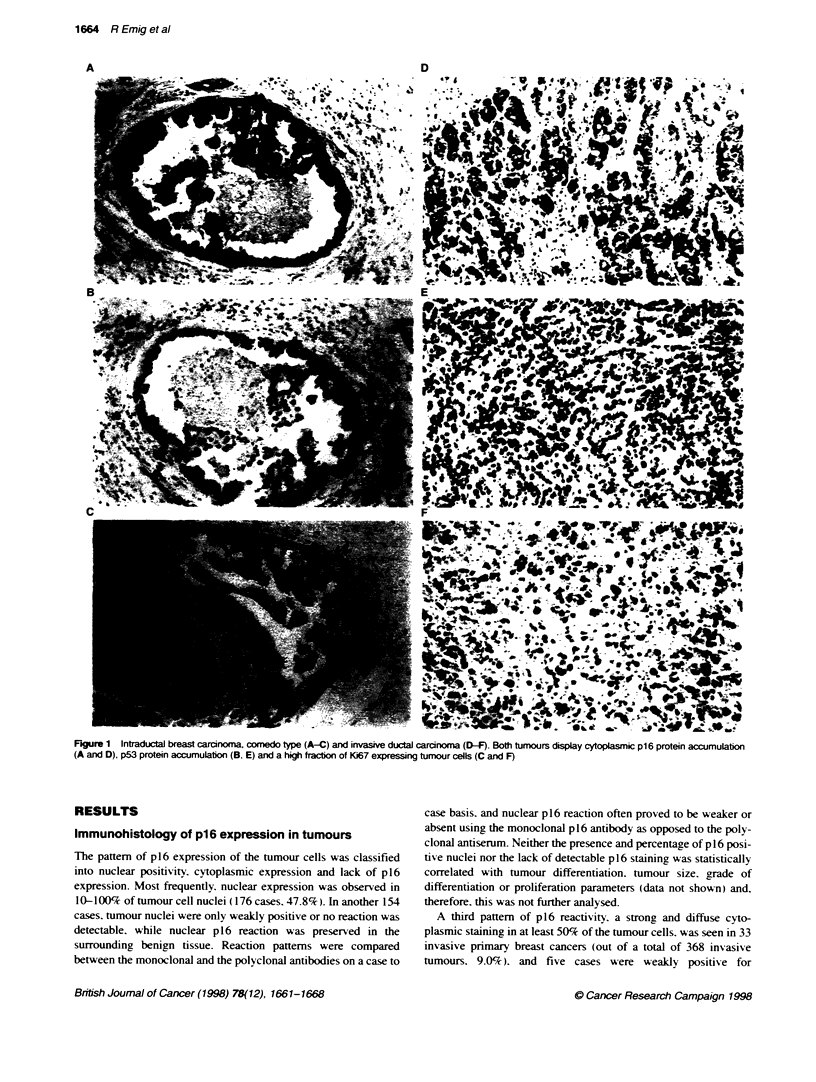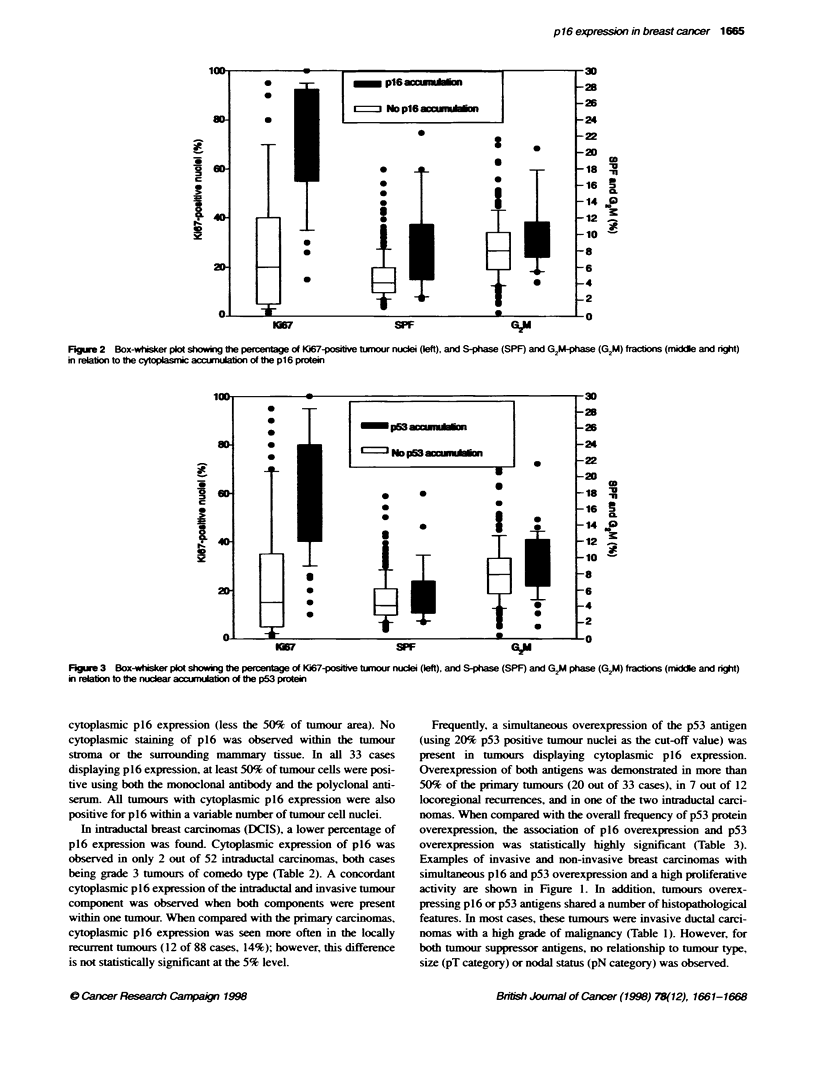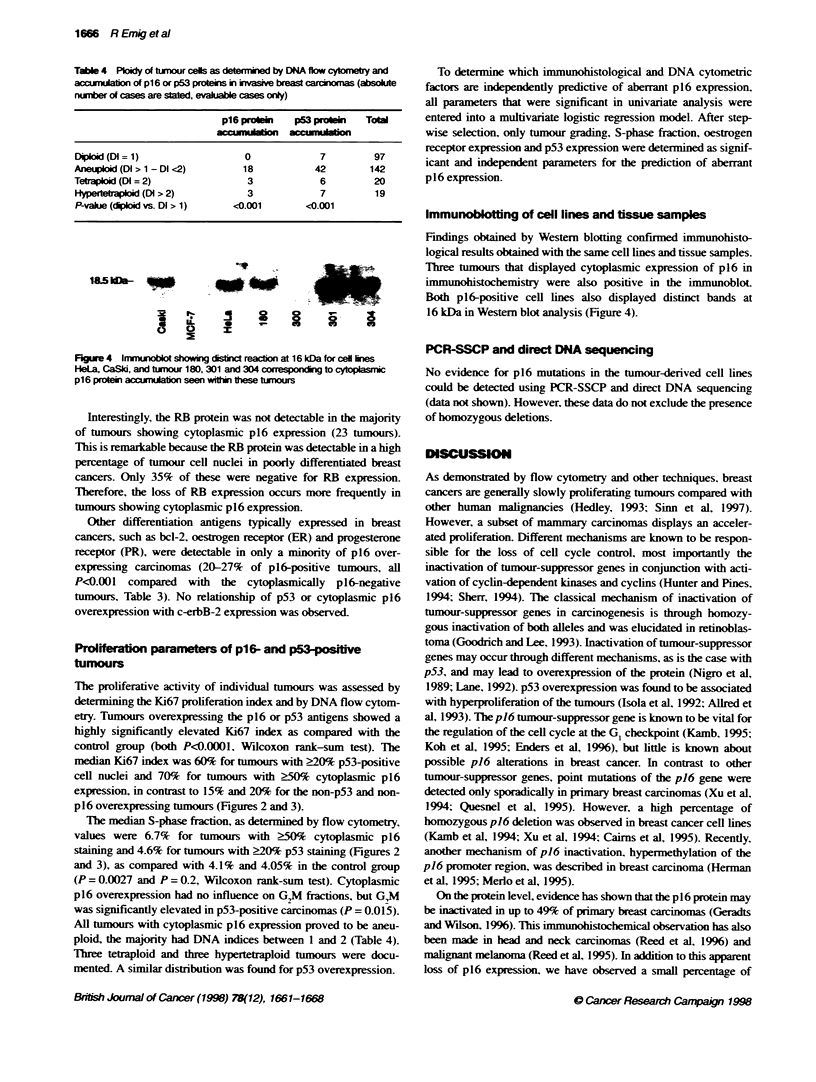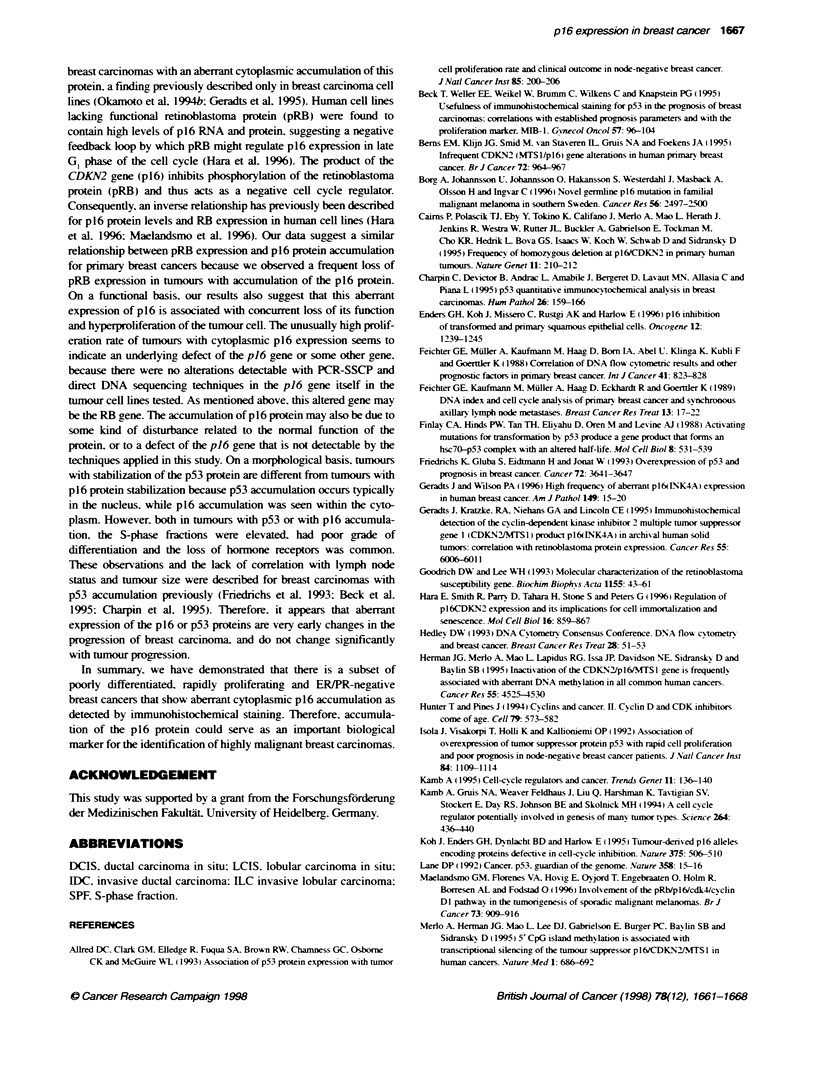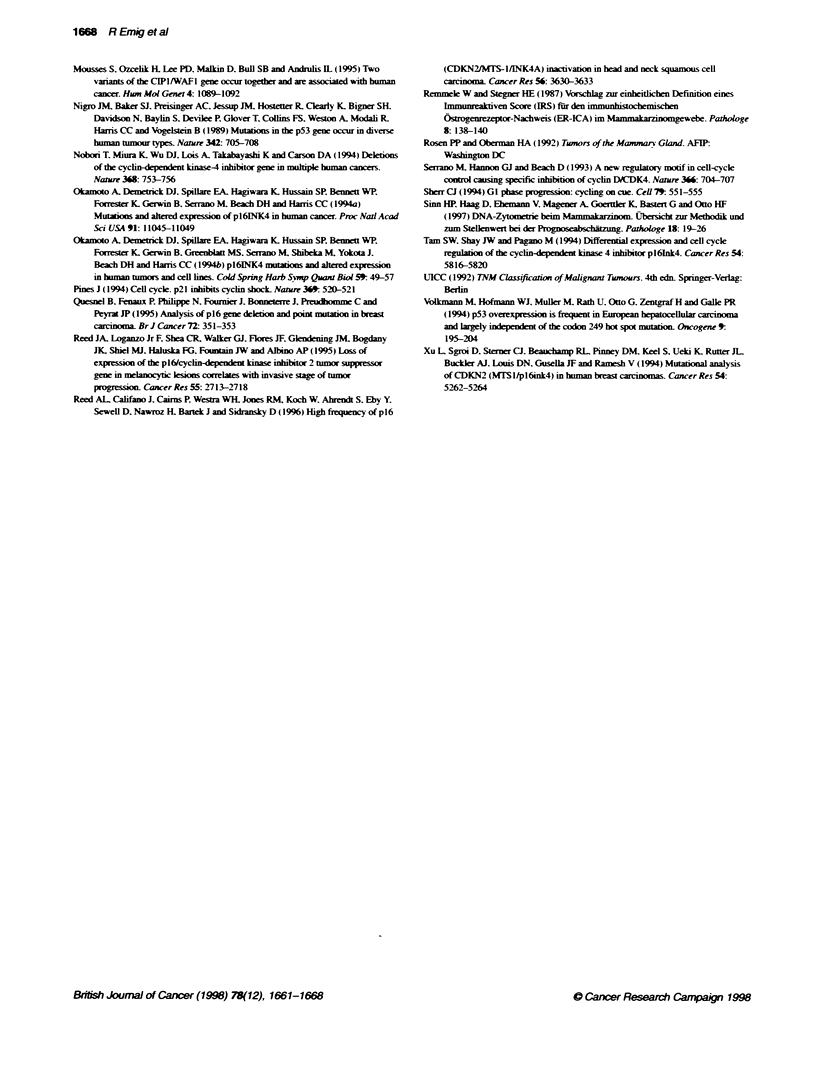# Aberrant cytoplasmic expression of the p16 protein in breast cancer is associated with accelerated tumour proliferation.

**DOI:** 10.1038/bjc.1998.739

**Published:** 1998-12

**Authors:** R. Emig, A. Magener, V. Ehemann, A. Meyer, F. Stilgenbauer, M. Volkmann, D. Wallwiener, H. P. Sinn

**Affiliations:** Frauenklinik, Tübingen, Germany.

## Abstract

**Images:**


British Journal of Cancer (1998) 78(12), 1661–1668
© 1998 Cancer Research Campaign

### Aberrant cytoplasmic expression of the p16 protein in breast cancer is associated with accelerated tumour proliferation

R Emig[1], A Magener[2], V Ehemann[1], A Meyer[2], F Stilgenbauer[3], M Volkmann[3], D Wallwiener[1] and H-P Sinn[2]

[1]Frauenklinik, Schleichstr. 4, 72076 Tübingen, Germany; [2]Pathologisches Institut, [3]Ludolf-Krehl-Klinik, der Universität Heidelberg, 69120 Heidelberg, Germany

**Summary** The p16 protein plays an important role in the transition of cells into the $G_1$ phase of the cell cycle. We have studied the prevalence of p16 protein expression in breast carcinomas in a prospective series of 368 invasive and 52 non-invasive malignancies, as well as in 88 locally recurring tumours and three tumour cell lines. p16 protein expression was evaluated immunohistochemically on paraffin sections using monoclonal and polyclonal anti-p16 antibodies, and by immunoblotting of tumour cell suspensions. Tumour cell lines were also subjected to polymerase chain reaction-single strand polymorphism (PCR-SSCP) analysis and direct DNA sequencing. The results were compared with established prognostic parameters, DNA flow cytometry and p53 protein expression. In 33 (9%) invasive and two (4%) intraductal carcinomas, a cytoplasmic accumulation of the p16 protein was seen. These cases were characterized by poor histological grade of differentiation, loss of of oestrogen receptors and progesterone receptors and frequent overexpression of the p53 protein. In addition, breast carcinomas with aberrant p16 expression demonstrated a high proliferative activity, with median S-phase fractions 74% higher than in the control group and the median Ki67 fractions elevated to 75%. A genetic alteration of the p16 gene was not detectable in three analysed cell lines with cytoplasmic p16 expression applying PCR-SSCP and direct DNA sequencing. These results indicate that cytoplasmic accumulation of the p16 protein identifies a subset of highly malignant breast carcinomas with accelerated tumour proliferation and other unfavourable parameters in breast cancer. The described protein accumulation is apparently not caused by an alteration of the *p16* gene.

**Keywords:** p16; breast cancer; proliferation; flow cytometry; immunohistochemistry; genetic alteration

The cell cycle is regulated by a number of cyclin-dependent kinases (CdKs). A number of different proteins inhibit the progression through the cell cycle by interference with the CdKs, a phenomenon especially interesting in malignant cells. Among these proteins are: p27, induced mainly by cell–cell contact; p21 (WAF-1, CIP1, CDI1), activated by the tumour-suppressor gene *p53* (Pines, 1994; Mousses et al, 1995); and p16 (CDKN2, MTS1), which inhibits CdK4.

*p16*, located on chromosome 9p21, is considered to play an important role as a tumour-suppressor gene (Serrano et al, 1993; Kamb et al, 1994). In cell lines of different tumour types, such as lung cancer, bladder tumours and lymphatic leukaemia, genetic changes in chromosome 9p21 have been described in 30–85% of cases (Nobori et al, 1994; Xu et al, 1994). In fresh solid tumours, the incidence of genetic alterations, such as the homozygous deletion of *p16*, is only in 10–20%. Some studies have been performed to elucidate the role of *p16* in breast cancer, applying polymerase chain reaction-single strand polymorphism (PCR-SSCP), Southern blot analysis and other techniques on cell lines and breast carcinoma samples (Kamb et al, 1994; Berns et al, 1995; Quesnel et al, 1995). It was concluded that gene alterations of *CDKN2* and the occurrence of genetic damage to the *CDKN2* gene in breast cancer are rare events and are not likely to be involved in the carcinogenesis and progression of breast cancer. However, a recently published report demonstrated that inactivation of *p16* occurs in 33% of breast cancer cell lines because of aberrant or de novo 5′CpG island methylation of *p16* (Herman et al, 1995).

Few data pertain to the immunohistochemical detection of p16 alterations in malignant cells. A loss of expression of p16 during the invasive stage of tumour progression was observed in melanocytic lesions (Reed et al, 1995).

Immunohistochemistry is an established method used to analyse expression and accumulation of many different proteins, including tumour-suppressor genes. In this context, accumulation of the p53 protein has been shown to be due to alterations of the gene (Finlay et al, 1988). Based on the knowledge of the role of *p16* and *p53* in cell cycle control, we have analysed both the p53 and p16 protein expression in this study and compared the results with the rate of tumour proliferation (as determined by flow cytometry and calculation of the Ki67 expression detected by immunocytochemistry). We further correlated these findings to established prognostic factors such as grading, tumour type, lymph node involvement, oestrogen and progesterone receptor status and other prognostic factors such as Bcl-2, c-ErbB-2 and retinoblastoma (RB), detected by immunohistochemistry. Our results indicate that in a subset of breast carcinomas overexpression of the p16 gene product is associated with rapid cell proliferation.

*Received 16 January 1998*
*Revised 18 March 1998*
*Accepted 24 March 1998*

*Correspondence to:* H-P Sinn, Pathologisches Institut, Universität Heidelberg, Im Neuenheimer Feld 220, 69120 Heidelberg, Germany

### MATERIALS AND METHODS

### Tumour specimens and immunohistochemical staining

Five hundred and twelve breast carcinomas from patients who were treated in 1995 and 1996 at the University Women's Hospital of

**Table 1** Tumour type, stage and histological grade of malignancy of invasive breast cancers studied, and protein accumulation of p16 and p53 (categories that are 'not applicable' are not listed; n.s. indicates *P*-values statistically not significant)

| | Total | p16 protein accumulation | p53 protein accumulation |
|---|---|---|---|
| Median age (years) | 53.7 | 52.2 | 55.2 |
| *P*-value | | n.s. | n.s. |
| Tumour type | | | |
| IDC | 292 | 28 | 58 |
| ILC | 48 | 2 | 5 |
| Medullary | 3 | 3 | 3 |
| Other (mucinous, papillary, tubular) | 25 | 0 | 1 |
| *P*-value (IDC vs. ILC) | | n.s. | n.s. |
| pT category | | | |
| pT1 | 170 | 18 | 27 |
| pT2 | 158 | 10 | 29 |
| pT3 | 17 | 1 | 3 |
| pT4 | 12 | 0 | 3 |
| *P*-value (pT1 vs pT2–4) | | n.s. | n.s. |
| pN category | | | |
| pN0 | 185 | 14 | 28 |
| pN1 | 136 | 14 | 28 |
| pN2 | 10 | 0 | 2 |
| *P*-value (pN0 vs. pN1,2) | | n.s. | n.s. |
| Tumour grading | | | |
| G1 | 56 | 0 | 0 |
| G2 | 180 | 3 | 16 |
| G3 | 119 | 25 | 45 |
| *P*-value (G1,2 vs. G3) | | <0.0001 | <0.0001 |

Heidelberg were examined, including 368 invasive primary cancers, 52 intraductal carcinomas (DCIS) and 88 locoregional tumour recurrences. All tumours were classified according to the AFIP classification for breast carcinomas (Rosen and Oberman, 1992) and staged according to the TNM system (UICC, 1992) (for details see Table 1). Tumour tissue was fixed in buffered formalin for at least 24 h, and subsequently embedded in paraffin and cut into tissue sections of approximately 2 μm. These sections were deparaffinized by incubation at 37°C for 24 h. After incubation at 50°C for another 30 min, the sections were immersed in xylene for 2 × 10 min, followed by rehydration through a graded series of ethanol (100%, 96%, 70% each for 5 min). Before immunohistochemical staining, the tissue sections underwent a microwaving procedure for 10 min at a high energy setting. After the addition of 50 ml of distilled water, an additional 7 min of high microwave energy was applied after addition of a diluted antigen retrieval buffer (Dako, Glostrup, Denmark). The slides were allowed to cool off at room temperature for a minimum of 20 min and sections stained using an automated immunohistochemical technique (Biotek TechMate, Biotek Solutions, Newport Beach, CA, USA) with strict adherence to the staining protocol. In brief, primary antibodies were applied for 30 min, followed by an indirect streptavidin biotin method with 30 min of secondary goat anti-mouse antibody and 45 min of streptavidin biotin conjugate. Primary antibodies used for detection of the p16 antigen were the mouse monoclonal antibody G175-405 (dilution 1:200, PharMingen, San Diego, CA, USA), and a rabbit polyclonal antibody (1:500, PharMingen). Other monoclonal antibodies used were anti-p53 antibody DO7 (1:100, Dako), anti-Ki67 antibody MIB1 (1:200, Dianova, Hamburg, Germany), anti-c-erbB-2 antibody 3B5 (1:2000, Oncogene Science, Manhasset, NY, USA), anti-bcl-2 antibody 124 (1:100, Dako) and anti-retinoblastoma antibody G3-245 (1:500, PharMingen). Oestrogen and progesterone receptors were detected with monoclonal antibody 1D5 for the oestrogen receptor and a polyclonal rabbit–anti-human antibody for the progesterone receptor (both 1:100, Dako).

### Microscopic evaluation of immunohistochemistry

The antibody staining was evaluated by two observers and recorded as the proportion of tumour cells positive for the evaluated antibodies. p16 expression was recorded separately as the percentage of positive tumour nuclei and, if present, an additional estimation of the percentage of tumour cells with cytoplasmic antigen expression. p53 expression and MiB1 (Ki67) staining were also registered as the percentage of nuclei staining strongly for the p53 or MiB1 proteins with regard to the total number of tumour nuclei in at least two high-power fields at the tumour invasion front. c-erbB-2 immunoreaction was scored from 0 to 4, considering merely cell membrane staining. Bcl-2 expression was considered positive if at least 15% of tumour cells were stained. Oestrogen and progesterone receptor staining was evaluated for staining intensity and number of positive nuclei (Remmele and Stegner, 1987), receptor positivity was assumed when the semi-quantitative score was at least three points (out of a maximum of 12 points).

### Flow cytometry

Tumour samples routinely underwent a cell cycle analysis using DNA flow cytometry as described elsewhere (Feichter et al, 1988, 1989). Briefly, tumour samples were thoroughly minced with scissors. The nuclei were extracted at room temperature by incubation in acid pepsin (3000 u $mg^{-1}$, Serva, Heidelberg, Germany) dissolved in 100 ml of 0.9 M sodium chloride containing 0.25% hydrochloric acid and carefully stirring for 20 min. After 30 s of sedimentation, 0.5 ml of the supernatant cell suspension was suspended in 1 μg $ml^{-1}$ 4′,6-diamindino-2-phenylindole (DAPI) dissolved in Tris-buffer pH 7.8. Minimum incubation time was 30 min. Flow cytometry was carried out using a PAS II flow cytometer equipped with a high-pressure mercury lamp (Partec, Münster, Germany) using the following filters: KG 1, BG 38 and UG 1 for excitation; TK 420 as dichroic mirror; and GG 435 as barrier filter. A flow rate of about 100 counts $s^{-1}$ was maintained by vacuum adjustment. DNA histograms of at least 10 000 counts were plotted. The DNA index of aneuploid cells was expressed as the relative modal DNA value of the aberrant peak in relation to the diploid one. Normal human lymphocytes with a coefficient of variation (CV) of 1.0 were used for calibration of the diploid peak.

The cell cycle–phase distribution patterns of the diploid and aneuploid tumours were calculated using the Multi-cycle software package (Phoenix Flow Systems, San Diego, CA, USA) after adjustments were made for debris and aggregation (nuclear doublets and triplets).

### Immunoblotting of tissue samples and cell lines

Cryopreserved breast cancer tissue samples were obtained from our tumour bank. Three tumours (nos 180, 301 and 304) were selected because of their known cytoplasmic expression of p16 as determined by paraffin immunohistochemistry. A fourth tumour (no. 300, not positive for p16 staining) was selected as a negative

© Cancer Research Campaign 1998

**Table 2** p16 and p53 protein accumulation in intraductal, invasive and recurrent breast cancer

| | p16 protein accumulation n (%) | p53 protein accumulation n (%) | Total |
|---|---|---|---|
| DCIS, non-comedo type | 0 | 5 (17) | 29 |
| DCIS, comedo type | 2 (9) | 9 (39) | 23 |
| Invasive carcinoma | 33 (9) | 82 (22) | 368 |
| Locoregional recurrence | 12 (14) | 29 (33) | 88 |

**Table 3** Correlation of p16 protein accumulation in invasive breast carcinomas with other immunohistochemical tumour markers (absolute numbers of cases are stated)

| | No p16 protein accumulation | p16 protein accumulation | P-value |
|---|---|---|---|
| p53 | | | |
| Negative (< 20% positive nuclei) | 286 | 15 | <0.0001 |
| Positive (≥ 20% positive nuclei) | 49 | 18 | |
| bcl-2 | | | |
| Negative (< 15% tumour cells) | 102 | 24 | <0.0001 |
| Positive (≥ 15% tumour cells) | 261 | 9 | |
| c-erbB-2 | | | |
| Negative (score 0,1) | 278 | 26 | 0.54 |
| Positive (score 2–4) | 57 | 7 | |
| Oestrogen receptors | | | |
| ER negative (score 0–3) | 72 | 24 | <0.0001 |
| ER positive (score 4–12) | 259 | 6 | |
| Progesterone receptors | | | |
| PR negative (score 0–3) | 129 | 25 | <0.0001 |
| PR positive (score 4–12) | 199 | 5 | |

control. Established tumour cell lines were also used. HeLa (human epithelioid cervical carcinoma cell line) and CaSki (human cervical epidermoid carcinoma cell line) as positive controls with known high amounts of p16 protein and MCF-7 (human breast adenocarcinoma cell line) as negative control (Tam et al. 1994).

Adherent growing cell lines were cultured in DMEM (Dulbecco's modified Eagle medium), harvested by washing in physiological phosphate-buffered saline (PBS), scraped into Falcon tubes after another application of 20 ml of PBS, and spun down at 1400 r.p.m. The pellet was transferred into lysis buffer containing 50 mmol of Tris-HCl pH 8.0, 150 mmol of sodium chloride, 1% NP 40 + pepstatin, pefablock and leupeptin. The samples were incubated on ice for 30 min followed by homogenization with a pestle for 10–12 times. Lysates were centrifuged at 14 000 r.p.m. at 5°C, and the supernatant used for immunoblotting.

Tissue samples from cryopreserved breast cancers (150–200 mg) were minced and homogenized in lysis buffer as specified above. Solid particles were sedimented, the supernatant was centrifuged at 14 000 r.p.m. at 5°C and also underwent gel electrophoresis.

One volume of protein homogenate was heated at 95°C for 10 min with two volumes of sample buffer containing β-mercaptoethanol. Equal amounts of protein were then subjected to electrophoresis in 12.5% sodium dodecyl sulphate (SDS) minigels at 20 mA. Proteins were transferred onto an Immobilon membrane (Millipore) in a Trans-Blot SD semi-dry transfer cell (Bio Rad) at 150 mA for 30 min. After blotting, the membrane was washed several times in PBS/0.05% Tween, and blocked for 1.5 h in PBS/0.05% Tween containing 0.2% bovine serum albumin (BSA). Subsequently, the membrane was incubated with primary polyclonal rabbit anti-human p16 antiserum (PharMingen, 1:1000, in 0.05% c-BSA/PBS/Tween) overnight. The secondary antibody (horseradish peroxidase-conjugated goat anti-rabbit IgG, Stratagene) was applied for 60 min, the membrane washed and treated according to the enhanced chemiluminescence (ECL) protocol (Boehringer, Mannheim, Germany). The radiographic film was exposed to the chemiluminescence membrane for up to 30 min.

### PCR-SSCP

Genomic DNA of three breast cancer cell lines established in our institution (two showing p16 protein accumulation) was isolated by detergent-mediated lysis and column chromatography as previously described (Volkmann et al. 1994). For controls, DNA preparations from different cell lines were used. HUH7 (hepatocellular carcinoma) and HepG2 (hepatoblastoma) have a p16 wild-type status. HL60 (human acute myeloid leukaemia), known to carry a point mutation in exon 2, and the line DLD1 (colon carcinoma) with a mutation in exon 1 of the *p16* gene served as mutant controls. Cell lines were obtained from DSM (Braunschweig, Germany). Exons 1 and 2 of the *p16* gene were screened for mutations by radioactive SSCP because no relevant mutations have yet been described in exon 3, which is only 11 bp large. PCR primers were used as described elsewhere (Okamoto et al. 1994*a*; Borg et al. 1996). PCR was carried out with incorporation of [$^{32}$P]dCTP (Hartmann Analytic, Braunschweig, Germany), with 5% DSMO and 0.1 mg ml$^{-1}$ BSA included. PCR conditions on the Perkin Elmer 480 Thermal cycler were: 35 cycles consisting of 92°C for 30 s, 58°C/62°C for 1 min, and 72°C for 1 min. Samples were denatured and underwent electrophoresis under non-denaturing conditions for autoradiography as previously described.

### Direct DNA sequencing

Direct DNA sequencing was performed on a Perkin-Elmer ABI-377 sequencer (ABI Prism) using a dye terminator cycle sequencing ready reaction kit (Perkin-Elmer, Weiterstadt, Germany). After purification using Quiaquick purification kit (Quiagen, Dassel, Germany), *p16* sequencing was performed using the same primers as for initial amplification.

### Statistics

Classified values were tested using the two-sided chi-squared test or Fisher's exact test for dichotomous variables. Continuous values were compared statistically by the Mann–Whitney rank–sum test. P-values of 0.05 or less were considered statistically significant. Multivariate analysis was performed by applying a logistic regression model and stepwise regression. All calculations were made with S-PLUS 3.3 (StatSci Division, MathSoft, Seattle, WA, USA).

© Cancer Research Campaign 1998 

**Figure 1** Intraductal breast carcinoma, comedo type (**A–C**) and invasive ductal carcinoma (**D–F**). Both tumours display cytoplasmic p16 protein accumulation (**A** and **D**), p53 protein accumulation (**B**, **E**) and a high fraction of Ki67 expressing tumour cells (**C** and **F**)

### RESULTS

### Immunohistology of p16 expression in tumours

The pattern of p16 expression of the tumour cells was classified into nuclear positivity, cytoplasmic expression and lack of p16 expression. Most frequently, nuclear expression was observed in 10–100% of tumour cell nuclei (176 cases, 47.8%). In another 154 cases, tumour nuclei were only weakly positive or no reaction was detectable, while nuclear p16 reaction was preserved in the surrounding benign tissue. Reaction patterns were compared between the monoclonal and the polyclonal antibodies on a case to case basis, and nuclear p16 reaction often proved to be weaker or absent using the monoclonal p16 antibody as opposed to the polyclonal antiserum. Neither the presence and percentage of p16 positive nuclei nor the lack of detectable p16 staining was statistically correlated with tumour differentiation, tumour size, grade of differentiation or proliferation parameters (data not shown) and, therefore, this was not further analysed.

A third pattern of p16 reactivity, a strong and diffuse cytoplasmic staining in at least 50% of the tumour cells, was seen in 33 invasive primary breast cancers (out of a total of 368 invasive tumours, 9.0%), and five cases were weakly positive for

© Cancer Research Campaign 1998

**Figure 2** Box-whisker plot showing the percentage of Ki67-positive tumour nuclei (left), and S-phase (SPF) and $G_2M$-phase ($G_2M$) fractions (middle and right) in relation to the cytoplasmic accumulation of the p16 protein

**Figure 3** Box-whisker plot showing the percentage of Ki67-positive tumour nuclei (left), and S-phase (SPF) and $G_2M$ phase ($G_2M$) fractions (middle and right) in relation to the nuclear accumulation of the p53 protein cytoplasmic p16 expression (less the 50% of tumour area). No cytoplasmic staining of p16 was observed within the tumour stroma or the surrounding mammary tissue. In all 33 cases displaying p16 expression, at least 50% of tumour cells were positive using both the monoclonal antibody and the polyclonal antiserum. All tumours with cytoplasmic p16 expression were also positive for p16 within a variable number of tumour cell nuclei.

In intraductal breast carcinomas (DCIS), a lower percentage of p16 expression was found. Cytoplasmic expression of p16 was observed in only 2 out of 52 intraductal carcinomas, both cases being grade 3 tumours of comedo type (Table 2). A concordant cytoplasmic p16 expression of the intraductal and invasive tumour component was observed when both components were present within one tumour. When compared with the primary carcinomas, cytoplasmic p16 expression was seen more often in the locally recurrent tumours (12 of 88 cases, 14%); however, this difference is not statistically significant at the 5% level.

Frequently, a simultaneous overexpression of the p53 antigen (using 20% p53 positive tumour nuclei as the cut-off value) was present in tumours displaying cytoplasmic p16 expression. Overexpression of both antigens was demonstrated in more than 50% of the primary tumours (20 out of 33 cases), in 7 out of 12 locoregional recurrences, and in one of the two intraductal carcinomas. When compared with the overall frequency of p53 protein overexpression, the association of p16 overexpression and p53 overexpression was statistically highly significant (Table 3). Examples of invasive and non-invasive breast carcinomas with simultaneous p16 and p53 overexpression and a high proliferative activity are shown in Figure 1. In addition, tumours overexpressing p16 or p53 antigens shared a number of histopathological features. In most cases, these tumours were invasive ductal carcinomas with a high grade of malignancy (Table 1). However, for both tumour suppressor antigens, no relationship to tumour type, size (pT category) or nodal status (pN category) was observed.

**Table 4** Ploidy of tumour cells as determined by DNA flow cytometry and accumulation of p16 or p53 proteins in invasive breast carcinomas (absolute number of cases are stated, evaluable cases only)

| | p16 protein accumulation | p53 protein accumulation | Total |
|---|---|---|---|
| Diploid (DI = 1) | 0 | 7 | 97 |
| Aneuploid (DI > 1 – DI <2) | 18 | 42 | 142 |
| Tetraploid (DI = 2) | 3 | 6 | 20 |
| Hypertetraploid (DI > 2) | 3 | 7 | 19 |
| *P*-value (diploid vs. DI > 1) | <0.001 | <0.001 | |

**Figure 4** Immunoblot showing distinct reaction at 16 kDa for cell lines HeLa, CaSki, and tumour 180, 301 and 304 corresponding to cytoplasmic p16 protein accumulation seen within these tumours

Interestingly, the RB protein was not detectable in the majority of tumours showing cytoplasmic p16 expression (23 tumours). This is remarkable because the RB protein was detectable in a high percentage of tumour cell nuclei in poorly differentiated breast cancers. Only 35% of these were negative for RB expression. Therefore, the loss of RB expression occurs more frequently in tumours showing cytoplasmic p16 expression.

Other differentiation antigens typically expressed in breast cancers, such as bcl-2, oestrogen receptor (ER) and progesterone receptor (PR), were detectable in only a minority of p16 overexpressing carcinomas (20–27% of p16-positive tumours, all $P<0.001$ compared with the cytoplasmically p16-negative tumours, Table 3). No relationship of p53 or cytoplasmic p16 overexpression with c-erbB-2 expression was observed.

### Proliferation parameters of p16- and p53-positive tumours

The proliferative activity of individual tumours was assessed by determining the Ki67 proliferation index and by DNA flow cytometry. Tumours overexpressing the p16 or p53 antigens showed a highly significantly elevated Ki67 index as compared with the control group (both $P<0.0001$, Wilcoxon rank–sum test). The median Ki67 index was 60% for tumours with ≥20% p53-positive cell nuclei and 70% for tumours with ≥50% cytoplasmic p16 expression, in contrast to 15% and 20% for the non-p53 and non-p16 overexpressing tumours (Figures 2 and 3).

The median S-phase fraction, as determined by flow cytometry, values were 6.7% for tumours with ≥50% cytoplasmic p16 staining and 4.6% for tumours with ≥20% p53 staining (Figures 2 and 3), as compared with 4.1% and 4.05% in the control group ($P = 0.0027$ and $P = 0.2$, Wilcoxon rank-sum test). Cytoplasmic p16 overexpression had no influence on $G_2M$ fractions, but $G_2M$ was significantly elevated in p53-positive carcinomas ($P = 0.015$). All tumours with cytoplasmic p16 expression proved to be aneuploid, the majority had DNA indices between 1 and 2 (Table 4). Three tetraploid and three hypertetraploid tumours were documented. A similar distribution was found for p53 overexpression.

To determine which immunohistological and DNA cytometric factors are independently predictive of aberrant p16 expression, all parameters that were significant in univariate analysis were entered into a multivariate logistic regression model. After stepwise selection, only tumour grading, S-phase fraction, oestrogen receptor expression and p53 expression were determined as significant and independent parameters for the prediction of aberrant p16 expression.

### Immunoblotting of cell lines and tissue samples

Findings obtained by Western blotting confirmed immunohistological results obtained with the same cell lines and tissue samples. Three tumours that displayed cytoplasmic expression of p16 in immunohistochemistry were also positive in the immunoblot. Both p16-positive cell lines also displayed distinct bands at 16 kDa in Western blot analysis (Figure 4).

### PCR-SSCP and direct DNA sequencing

No evidence for p16 mutations in the tumour-derived cell lines could be detected using PCR-SSCP and direct DNA sequencing (data not shown). However, these data do not exclude the presence of homozygous deletions.

### DISCUSSION

As demonstrated by flow cytometry and other techniques, breast cancers are generally slowly proliferating tumours compared with other human malignancies (Hedley, 1993; Sinn et al, 1997). However, a subset of mammary carcinomas displays an accelerated proliferation. Different mechanisms are known to be responsible for the loss of cell cycle control, most importantly the inactivation of tumour-suppressor genes in conjunction with activation of cyclin-dependent kinases and cyclins (Hunter and Pines, 1994; Sherr, 1994). The classical mechanism of inactivation of tumour-suppressor genes in carcinogenesis is through homozygous inactivation of both alleles and was elucidated in retinoblastoma (Goodrich and Lee, 1993). Inactivation of tumour-suppressor genes may occur through different mechanisms, as is the case with *p53*, and may lead to overexpression of the protein (Nigro et al, 1989; Lane, 1992). p53 overexpression was found to be associated with hyperproliferation of the tumours (Isola et al, 1992; Allred et al, 1993). The *p16* tumour-suppressor gene is known to be vital for the regulation of the cell cycle at the $G_1$ checkpoint (Kamb, 1995; Koh et al, 1995; Enders et al, 1996), but little is known about possible *p16* alterations in breast cancer. In contrast to other tumour-suppressor genes, point mutations of the *p16* gene were detected only sporadically in primary breast carcinomas (Xu et al, 1994; Quesnel et al, 1995). However, a high percentage of homozygous *p16* deletion was observed in breast cancer cell lines (Kamb et al, 1994; Xu et al, 1994; Cairns et al, 1995). Recently, another mechanism of *p16* inactivation, hypermethylation of the *p16* promoter region, was described in breast carcinoma (Herman et al, 1995; Merlo et al, 1995).

On the protein level, evidence has shown that the p16 protein may be inactivated in up to 49% of primary breast carcinomas (Geradts and Wilson, 1996). This immunohistochemical observation has also been made in head and neck carcinomas (Reed et al, 1996) and malignant melanoma (Reed et al, 1995). In addition to this apparent loss of p16 expression, we have observed a small percentage of

© Cancer Research Campaign 1998

breast carcinomas with an aberrant cytoplasmic accumulation of this protein, a finding previously described only in breast carcinoma cell lines (Okamoto et al. 1994*b*; Geradts et al. 1995). Human cell lines lacking functional retinoblastoma protein (pRB) were found to contain high levels of p16 RNA and protein, suggesting a negative feedback loop by which pRB might regulate p16 expression in late $G_1$ phase of the cell cycle (Hara et al. 1996). The product of the *CDKN2* gene (p16) inhibits phosphorylation of the retinoblastoma protein (pRB) and thus acts as a negative cell cycle regulator. Consequently, an inverse relationship has previously been described for p16 protein levels and RB expression in human cell lines (Hara et al. 1996; Maelandsmo et al. 1996). Our data suggest a similar relationship between pRB expression and p16 protein accumulation for primary breast cancers because we observed a frequent loss of pRB expression in tumours with accumulation of the p16 protein. On a functional basis, our results also suggest that this aberrant expression of p16 is associated with concurrent loss of its function and hyperproliferation of the tumour cell. The unusually high proliferation rate of tumours with cytoplasmic p16 expression seems to indicate an underlying defect of the *p16* gene or some other gene, because there were no alterations detectable with PCR-SSCP and direct DNA sequencing techniques in the *p16* gene itself in the tumour cell lines tested. As mentioned above, this altered gene may be the RB gene. The accumulation of p16 protein may also be due to some kind of disturbance related to the normal function of the protein, or to a defect of the *p16* gene that is not detectable by the techniques applied in this study. On a morphological basis, tumours with stabilization of the p53 protein are different from tumours with p16 protein stabilization because p53 accumulation occurs typically in the nucleus, while p16 accumulation was seen within the cytoplasm. However, both in tumours with p53 or with p16 accumulation, the S-phase fractions were elevated, had poor grade of differentiation and the loss of hormone receptors was common. These observations and the lack of correlation with lymph node status and tumour size were described for breast carcinomas with p53 accumulation previously (Friedrichs et al. 1993; Beck et al. 1995; Charpin et al. 1995). Therefore, it appears that aberrant expression of the p16 or p53 proteins are very early changes in the progression of breast carcinoma, and do not change significantly with tumour progression.

In summary, we have demonstrated that there is a subset of poorly differentiated, rapidly proliferating and ER/PR-negative breast cancers that show aberrant cytoplasmic p16 accumulation as detected by immunohistochemical staining. Therefore, accumulation of the p16 protein could serve as an important biological marker for the identification of highly malignant breast carcinomas.

### ACKNOWLEDGEMENT

This study was supported by a grant from the Forschungsförderung der Medizinischen Fakultät, University of Heidelberg, Germany.

### ABBREVIATIONS

DCIS, ductal carcinoma in situ; LCIS, lobular carcinoma in situ; IDC, invasive ductal carcinoma; ILC invasive lobular carcinoma; SPF, S-phase fraction.

### REFERENCES

Allred DC, Clark GM, Elledge R, Fuqua SA, Brown RW, Chamness GC, Osborne CK and McGuire WL (1993) Association of p53 protein expression with tumor cell proliferation rate and clinical outcome in node-negative breast cancer. *J Natl Cancer Inst* **85**: 200–206

Beck T, Weller EE, Weikel W, Brumm C, Wilkens C and Knapstein PG (1995) Usefulness of immunohistochemical staining for p53 in the prognosis of breast carcinomas: correlations with established prognosis parameters and with the proliferation marker, MIB-1. *Gynecol Oncol* **57**: 96–104

Berns EM, Klijn JG, Smid M, van Staveren IL, Gruis NA and Foekens JA (1995) Infrequent CDKN2 (MTS1/p16) gene alterations in human primary breast cancer. *Br J Cancer* **72**: 964–967

Borg A, Johannsson U, Johannsson O, Hakansson S, Westerdahl J, Masback A, Olsson H and Ingvar C (1996) Novel germline p16 mutation in familial malignant melanoma in southern Sweden. *Cancer Res* **56**: 2497–2500

Cairns P, Polascik TJ, Eby Y, Tokino K, Califano J, Merlo A, Mao L, Herath J, Jenkins R, Westra W, Rutter JL, Buckler A, Gabrielson E, Tockman M, Cho KR, Hedrik L, Bova GS, Isaacs W, Koch W, Schwab D and Sidransky D (1995) Frequency of homozygous deletion at p16/CDKN2 in primary human tumours. *Nature Genet* **11**: 210–212

Charpin C, Devictor B, Andrac L, Amabile J, Bergeret D, Lavaut MN, Allasia C and Piana L (1995) p53 quantitative immunocytochemical analysis in breast carcinomas. *Hum Pathol* **26**: 159–166

Enders GH, Koh J, Missero C, Rustgi AK and Harlow E (1996) p16 inhibition of transformed and primary squamous epithelial cells. *Oncogene* **12**: 1239–1245

Feichter GE, Müller A, Kaufmann M, Haag D, Born IA, Abel U, Klinga K, Kubli F and Goerttler K (1988) Correlation of DNA flow cytometric results and other prognostic factors in primary breast cancer. *Int J Cancer* **41**: 823–828

Feichter GE, Kaufmann M, Müller A, Haag D, Eckhardt R and Goerttler K (1989) DNA index and cell cycle analysis of primary breast cancer and synchronous axillary lymph node metastases. *Breast Cancer Res Treat* **13**: 17–22

Finlay CA, Hinds PW, Tan TH, Eliyahu D, Oren M and Levine AJ (1988) Activating mutations for transformation by p53 produce a gene product that forms an hsc70–p53 complex with an altered half-life. *Mol Cell Biol* **8**: 531–539

Friedrichs K, Gluba S, Eidtmann H and Jonat W (1993) Overexpression of p53 and prognosis in breast cancer. *Cancer* **72**: 3641–3647

Geradts J and Wilson PA (1996) High frequency of aberrant p16(INK4A) expression in human breast cancer. *Am J Pathol* **149**: 15–20

Geradts J, Kratzke, RA, Niehans GA and Lincoln CE (1995) Immunohistochemical detection of the cyclin-dependent kinase inhibitor 2 multiple tumor suppressor gene 1 (CDKN2/MTS1) product p16(INK4A) in archival human solid tumors: correlation with retinoblastoma protein expression. *Cancer Res* **55**: 6006–6011

Goodrich DW and Lee WH (1993) Molecular characterization of the retinoblastoma susceptibility gene. *Biochim Biophys Acta* **1155**: 43–61

Hara E, Smith R, Parry D, Tahara H, Stone S and Peters G (1996) Regulation of p16CDKN2 expression and its implications for cell immortalization and senescence. *Mol Cell Biol* **16**: 859–867

Hedley DW (1993) DNA Cytometry Consensus Conference. DNA flow cytometry and breast cancer. *Breast Cancer Res Treat* **28**: 51–53

Herman JG, Merlo A, Mao L, Lapidus RG, Issa JP, Davidson NE, Sidransky D and Baylin SB (1995) Inactivation of the CDKN2/p16/MTS1 gene is frequently associated with aberrant DNA methylation in all common human cancers. *Cancer Res* **55**: 4525–4530

Hunter T and Pines J (1994) Cyclins and cancer. II. Cyclin D and CDK inhibitors come of age. *Cell* **79**: 573–582

Isola J, Visakorpi T, Holli K and Kallioniemi OP (1992) Association of overexpression of tumor suppressor protein p53 with rapid cell proliferation and poor prognosis in node-negative breast cancer patients. *J Natl Cancer Inst* **84**: 1109–1114

Kamb A (1995) Cell-cycle regulators and cancer. *Trends Genet* **11**: 136–140

Kamb A, Gruis NA, Weaver Feldhaus J, Liu Q, Harshman K, Tavtigian SV, Stockert E, Day RS, Johnson BE and Skolnick MH (1994) A cell cycle regulator potentially involved in genesis of many tumor types. *Science* **264**: 436–440

Koh J, Enders GH, Dynlacht BD and Harlow E (1995) Tumour-derived p16 alleles encoding proteins defective in cell-cycle inhibition. *Nature* **375**: 506–510

Lane DP (1992) Cancer. p53, guardian of the genome. *Nature* **358**: 15–16

Maelandsmo GM, Florenes VA, Hovig E, Oyjord T, Engebraaten O, Holm R, Borresen AL and Fodstad O (1996) Involvement of the pRb/p16/cdk4/cyclin D1 pathway in the tumorigenesis of sporadic malignant melanomas. *Br J Cancer* **73**: 909–916

Merlo A, Herman JG, Mao L, Lee DJ, Gabrielson E, Burger PC, Baylin SB and Sidransky D (1995) 5′ CpG island methylation is associated with transcriptional silencing of the tumour suppressor p16/CDKN2/MTS1 in human cancers. *Nature Med* **1**: 686–692

© Cancer Research Campaign 1998

Mousses S, Ozcelik H, Lee PD, Malkin D, Bull SB and Andrulis IL (1995) Two variants of the CIP1/WAF1 gene occur together and are associated with human cancer. *Hum Mol Genet* **4**: 1089–1092

Nigro JM, Baker SJ, Preisinger AC, Jessup JM, Hostetter R, Clearly K, Bigner SH, Davidson N, Baylin S, Devilee P, Glover T, Collins FS, Weston A, Modali R, Harris CC and Vogelstein B (1989) Mutations in the p53 gene occur in diverse human tumour types. *Nature* **342**: 705–708

Nobori T, Miura K, Wu DJ, Lois A, Takabayashi K and Carson DA (1994) Deletions of the cyclin-dependent kinase-4 inhibitor gene in multiple human cancers. *Nature* **368**: 753–756

Okamoto A, Demetrick DJ, Spillare EA, Hagiwara K, Hussain SP, Bennett WP, Forrester K, Gerwin B, Serrano M, Beach DH and Harris CC (1994*a*) Mutations and altered expression of p16INK4 in human cancer. *Proc Natl Acad Sci USA* **91**: 11045–11049

Okamoto A, Demetrick DJ, Spillare EA, Hagiwara K, Hussain SP, Bennett WP, Forrester K, Gerwin B, Greenblatt MS, Serrano M, Shibeka M, Yokota J, Beach DH and Harris CC (1994*b*) p16INK4 mutations and altered expression in human tumors and cell lines. *Cold Spring Harb Symp Quant Biol* **59**: 49–57

Pines J (1994) Cell cycle. p21 inhibits cyclin shock. *Nature* **369**: 520–521

Quesnel B, Fenaux P, Philippe N, Fournier J, Bonneterre J, Preudhomme C and Peyrat JP (1995) Analysis of p16 gene deletion and point mutation in breast carcinoma. *Br J Cancer* **72**: 351–353

Reed JA, Loganzo Jr F, Shea CR, Walker GJ, Flores JF, Glendening JM, Bogdany JK, Shiel MJ, Haluska FG, Fountain JW and Albino AP (1995) Loss of expression of the p16/cyclin-dependent kinase inhibitor 2 tumor suppressor gene in melanocytic lesions correlates with invasive stage of tumor progression. *Cancer Res* **55**: 2713–2718

Reed AL, Califano J, Cairns P, Westra WH, Jones RM, Koch W, Ahrendt S, Eby Y, Sewell D, Nawroz H, Bartek J and Sidransky D (1996) High frequency of p16 (CDKN2/MTS-1/INK4A) inactivation in head and neck squamous cell carcinoma. *Cancer Res* **56**: 3630–3633

Remmele W and Stegner HE (1987) Vorschlag zur einheitlichen Definition eines Immunreaktiven Score (IRS) für den immunhistochemischen Östrogenrezeptor-Nachweis (ER-ICA) im Mammakarzinomgewebe. *Pathologe* **8**: 138–140

Rosen PP and Oberman HA (1992) *Tumors of the Mammary Gland*. AFIP: Washington DC

Serrano M, Hannon GJ and Beach D (1993) A new regulatory motif in cell-cycle control causing specific inhibition of cyclin D/CDK4. *Nature* **366**: 704–707

Sherr CJ (1994) G1 phase progression: cycling on cue. *Cell* **79**: 551–555

Sinn HP, Haag D, Ehemann V, Magener A, Goerttler K, Bastert G and Otto HF (1997) DNA-Zytometrie beim Mammakarzinom. Übersicht zur Methodik und zum Stellenwert bei der Prognoseabschätzung. *Pathologe* **18**: 19–26

Tam SW, Shay JW and Pagano M (1994) Differential expression and cell cycle regulation of the cyclin-dependent kinase 4 inhibitor p16Ink4. *Cancer Res* **54**: 5816–5820

UICC (1992) *TNM Classification of Malignant Tumours*. 4th edn. Springer-Verlag: Berlin

Volkmann M, Hofmann WJ, Muller M, Rath U, Otto G, Zentgraf H and Galle PR (1994) p53 overexpression is frequent in European hepatocellular carcinoma and largely independent of the codon 249 hot spot mutation. *Oncogene* **9**: 195–204

Xu L, Sgroi D, Sterner CJ, Beauchamp RL, Pinney DM, Keel S, Ueki K, Rutter JL, Buckler AJ, Louis DN, Gusella JF and Ramesh V (1994) Mutational analysis of CDKN2 (MTS1/p16ink4) in human breast carcinomas. *Cancer Res* **54**: 5262–5264

© Cancer Research Campaign 1998